# Grape Pomace and Ferulic Acid Improve Antioxidant Enzyme Activity and Gut Histomorphometry in Heat-Stressed Finishing Pigs

**DOI:** 10.3390/ani15162382

**Published:** 2025-08-13

**Authors:** María A. Ospina-Romero, Leslie S. Medrano-Vázquez, Araceli Pinelli-Saavedra, Miguel Ángel Barrera-Silva, Martín Valenzuela-Melendres, Miguel Ángel Martínez-Téllez, Reyna Fabiola Osuna-Chávez, María del Refugio Robles-Burgueño, Humberto González-Rios

**Affiliations:** 1 Centro de Investigación en Alimentación y Desarrollo, A.C. Carretera Gustavo Enrique Astiazarán Rosas, No. 46, Col. La Victoria, Hermosillo 83304, Sonora, Mexico; mospina221@estudiantes.ciad.mx (M.A.O.-R.); lmedrano224@estudiantes.ciad.mx (L.S.M.-V.); pinelli@ciad.mx (A.P.-S.); martin@ciad.mx (M.V.-M.); norawa@ciad.mx (M.Á.M.-T.); cuquis@ciad.mx (M.d.R.R.-B.); 2 Departamento de Agricultura y Ganadería, Universidad de Sonora, Carretera a Bahía de Kino km 21, Hermosillo 83000, Sonora, Mexico; miguel.barrera@unison.mx (M.Á.B.-S.); reyna.osuna@unison.mx (R.F.O.-C.)

**Keywords:** phenolic compounds, functional additive, phytogenic, finishing pigs, intestinal health, heat stress

## Abstract

Given the restrictions on animal growth promoters, alternative plant-based additives—particularly those rich in phenolic compounds, such as agro-industrial by-products—have been explored. These additives help to mitigate heat stress, which negatively affects productivity by impacting intestinal health and antioxidant status. This study evaluated the effects of individual and combined supplementation of ferulic acid (FA) and grape pomace (GP) on antioxidant enzyme activity, as well as intestinal histomorphometry, in finishing pigs under heat stress. Forty Yorkshire × Duroc pigs were randomly assigned to four treatments: control, 25 mg/kg FA, 2.5% GP, and MIX (FA + GP). FA supplementation increased intestinal villus height, while GP increased villus width in the duodenum and jejunum (*p* < 0.05). Antioxidant enzyme activity (SOD, CAT, and GPx) increased in pigs supplemented with GP (*p* < 0.05). These results suggest that GP and FA have potential as functional additives in monogastric diets, improving intestinal health and muscle antioxidant status and contributing to growth modulation.

## Simple Summary

Pigs are highly susceptible to heat stress, which can negatively impact their health and performance. The inclusion of phenolic compounds in monogastric diets has been considered a potential alternative to mitigate the impact of this condition on the animals. This study investigated how individual or combined dietary supplementation with ferulic acid and grape pomace (an agro-industrial by-product rich in phenolic compounds) can improve antioxidant enzyme activity and intestinal villus health in pigs exposed to heat stress conditions. The inclusion of grape pomace improved antioxidant enzyme activity in the muscles and increased villus width in both the duodenum and jejunum, while the inclusion of ferulic acid increased villus height. The findings suggest that both additives can improve the antioxidant status and intestinal health of finishing pigs.

## 1. Introduction

Stressful stimuli, such as sudden temperature rises and high humidity during the summer, exacerbate heat stress in finishing pig production systems (temperatures exceeding 30–35 °C), representing a significant threat and ongoing challenge in pig farming [1]. These conditions promote oxidative stress processes, which negatively affect growth, intestinal health, and antioxidant and immune systems in pigs [1,2]. Consequently, these effects impact both productive performance (caused by the reduction in feed efficiency due to the inhibition of feed consumption or due to poor absorption of nutrients) and animal welfare (hyperthermia, dysbiosis, hormonal disorders, immunosuppression, and behavioral disturbances), generating significant economic losses for producers and the pork industry [3,4,5]. In this sense, previous research has shown that acute and chronic heat stress negatively impact the intestinal barrier’s functionality, morphology, and integrity in finishing pigs [6]. These adverse effects compromise the intestine’s protective function, as well as its role in the digestion and absorption of nutrients, which are key determinants of final productive performance. At the same time, these repercussions of heat stress also contribute to significant damage to skeletal muscle, one of the tissues involved in energy metabolism, whose antioxidant system plays a crucial role in mitigating the effects of heat stress. In response to this situation, the activities of antioxidant enzymes, such as superoxide dismutase (SOD), catalase (CAT), and glutathione peroxidase (GPx), among others, may increase or decrease depending on the magnitude and duration of exposure to the stressor [7,8]. However, the antioxidant status in animals is also affected. For this reason, efforts have been made to improve the endogenous response in monogastric animals by supplementing them with various antioxidant sources [9]. Therefore, the inclusion of phenolic compounds (PCs) in monogastric diets has been evaluated and proposed as a promising nutritional strategy to counteract or mitigate the impacts of heat stress on pigs and maintain their productive performance when they are outside their thermoneutral zone [10]. The particular interest in these secondary metabolites lies in their potential to positively impact animal performance and health due to their biological properties, such as their antioxidant, anti-inflammatory, antibacterial, immunomodulatory, antiproliferative, and phytogenic activities, through various mechanisms that have been partially or fully elucidated [11,12,13]. Therefore, agro-industrial by-products with a diverse profile of PCs, such as grape pomace (GP), and pure compounds, such as ferulic acid (FA), have received significant attention in the area of animal nutrition to be used as attractive functional additives in finishing pig diets [14]. This interest is due to previous studies with pigs, which have shown that including this matrix and these metabolites improves intestinal barrier integrity, morphology, functionality, and nutrient digestibility which, in turn, contributes to improving the antioxidant status in various animal tissues [6,15]. These beneficial effects could be associated with growth and carcass quality changes, as previously observed in a recent study with pigs [16]. Similarly, in the search for new functional additives for poultry and pigs, it has been hypothesized that mixing PC-rich sources could promote synergistic effects and, thus, enhance beneficial effects as a growth modulator, which has been related to improvements in antioxidant activity and intestinal health. Therefore, this research aimed to evaluate the effects of the individual or combined supplementation of GP and FA on the activity of antioxidant enzymes in muscles and the histomorphometric characteristics of the intestinal tissue in finishing pigs raised under adverse heat stress conditions.

## 2. Materials and Methods

### 2.1. Preparation of Grape Pomace and Extracts and Identification of Phenolic Compounds

This study utilized a meal made from Tempranillo grape pomace, with the drying, grinding, and preservation processes carried out following the methodology previously employed by Ospina Romero et al. [16]. Subsequently, methanolic extracts were obtained from this by-product following a previously standardized methodology, with slight modifications [11] to the original technique [17]. The antioxidant capacity was determined using the FRAP, ABTS, and DPPH methods, along with the contents of soluble phenolic compounds (Folin–Ciocalteu), flavonoids, and both hydrolyzed and condensed tannins, as reported in the previous and complementary publication to this study [16].

The methanolic extracts were centrifuged at 10,000 rpm for 10 min using a Thermo Sorvall Scientific ST40r centrifuge (Thermo Fisher, Walham, MA, USA) and filtered through 0.2 µm membranes (Acrodisc Cytiva 4522, Thermo Fissher Scientific, Walham, MA, USA). Following this, phenolic acids and flavonoids were separated and fractionated according to the methodology proposed by Chen et al. [18]. In the resulting fractions (phenolic acids, flavonoids, and washing), the contents of gallic acid, protocatechuic acid, catechin, epicatechin, and quercetin were quantified.

Furthermore, these compounds were identified using high-performance liquid chromatography (HPLC) with an Agilent 1200, Infinity 1260 series system (Agilent Technologies, Santa Clara, CA, USA) equipped with a Nucleosil C18 column (5 µm, 150 mm × 4.6 mm, SUPELCO, Sigma-Aldrich, Darmstadt, Germany) and featuring a UV diode array and autosampler. The mobile phase consisted of water with 1% formic acid (phase A), while the organic phase (phase B) was made with acetonitrile and 1% formic acid. The elution gradient was performed with these solvents for a total period of 32 min (0, 5.5, 9.0, 16, 20, 31, and 32 min). The proportion of solvent A increased over time (0.8%, 6.8%, 10%, 20%, 25%, 100%, and 100%), while the proportion of solvent B decreased as the runtime progressed (99.2%, 93.2% 90%, 80%, 75%, 0%, and 0%). The flow rate of the mobile phase was set at 0.8 mL/min. A total of 19 standards of phenolic compounds were utilized, including gallic acid, chlorogenic acid, caffeic acid, gentisic acid, protocatechinic acid, syringic acid, synaptic acid, trans-cinnamic acid, ferulic acid, isoferulic acid, O-coumaric acid, P-coumaric acid, myricetin, quercetin, rutin, catechin, epicatechin, resveratrol, and vanillin (Sigma Aldrich, St. Louis, MO, USA). The retention times and spectral characteristics of both the standards and the phenolic compounds present were analyzed to determine the profile of compounds in grape pomace [19], with observations made at various wavelengths (260 nm, 280 nm, 320 nm, 370 nm, and 520 nm).

### 2.2. Feeding Trial in Finishing Pigs and Characterization of Experimental Diets

This study had the approval and supervision of the Universidad de Sonora Ethics Committee (CEI-UNISON/1A-3-01/2024). Moreover, all experimental procedures involving supplementation, handling, and slaughter complied with official Mexican animal welfare standards (NOM-033-SAG/ZOO, 2014; NOM-051-ZOO, 1995). The feeding trial was carried out in the pig experimental unit of the Department of Agriculture and Livestock (DAL) of the Universidad de Sonora during the summer (May/June) with temperature and humidity in the range of 29.9 ± 2.01 °C and 38.6 ± 8.3%, respectively. Therefore, the temperature humidity index (THI) ranged from 68.67 to 96.46 units, averaging 76.02 units, during the experimental period. The animals remained in a state of alertness under these THI values, reflecting that they were exposed to heat stress [16]. They presented an average rectal temperature value and an average respiratory frequency of 39.3 °C and 80 bpm, which exceeded the normal physiological values reported for the species.

For supplementation, the previously characterized GP [11] and ferulic acid (95% purity; feed-grade), supplied by Laboratorios Minkab S.A de C.V (Guadalajara, Jalisco, Mexico), were used. Forty male commercial crossbred pigs (Duroc × Yorkshire) with an average live weight of 80.2 kg and age of 19 weeks were used. The animals were individually housed in cages (0.6 × 2 m) and had ad libitum access to feeders and drinkers. Of the total group, 10 cages were randomly distributed under a completely randomized design with a 2 × 2 factorial arrangement in the following 4 treatments: control (a basal diet (BD) without additives); FA (BD + 25 mg FA/kg feed); GP (BD + 2.5% GP/kg); and MIX (BD + 25 mg FA + 2.5% GP/kg). The FA concentration tested was established based on a previous study by our working group [14], in which favorable and significant results were obtained in the productive performance of finishing pigs supplemented with 25 mg FA/kg feed. The inclusion percentage of grape pomace was selected according to the total phenol content estimated in this by-product and did not exceed 1500 mg/kg feed of phenolic compounds per animal/day, according to the recommendation of Mahfuz et al. [20], to not affect the animals’ feed intake. The experimental diets were previously characterized (the total PC content and antioxidant capacity) and were fed for 31 days during the pigs’ finishing stage. The basal diet (Supplementary Table S1) for the finishing stage consisted of 76.25% wheat grain, 17% soybean meal, 4.4% vegetable oil, and 2.4% amino acid, vitamin, and mineral premix, as evaluated in our previous study [16]. The diet contained 11.9% moisture, 7% fat, 2% crude fiber, and 7% ash, providing 14% crude protein and 3.35 Mcal/kg of net energy.

### 2.3. Muscle Sample Collection

Once the experimental period was over, pigs were slaughtered after 16 h of fasting; this process was carried out according to the regulations (NOM-033-ZOO-1995) at the slaughterhouse of the DAL of the Universidad de Sonora. The pigs were electrically stunned before bleeding. After the slaughter and evisceration process of the animals, at 45 min postmortem, samples of the longissimus thoracis muscle (2 × 1 × 1 cm) were taken from the right side of the carcass of 5 animals per treatment in triplicate (the eighth thoracic vertebrae). The samples were immediately frozen in liquid nitrogen and then transported to the Food and Development Research Center, where they were stored at −80 °C until fur.

### 2.4. Measurement of Antioxidant Enzyme Activity in Longissimus thoracis Muscle

Antioxidant enzyme activity was assessed in crude muscle extracts using a method with slight modifications to the original procedure [21,22]. Extracts were prepared from 100 mg of muscle in 3 volumes of sodium phosphate buffer (40 mM; pH 7.1) and homogenized for 30 s (T 25 Ultra-Turrax, IKA, Staufen, Germany). During homogenization, the samples were kept on ice. Subsequently, the homogenates were centrifuged (6400× *g*; 15 min; 4 °C).

The supernatants of the extracts were taken, divided into aliquots, and stored until analysis at −80 °C. The protein concentration of the extracts was determined using Bradford’s method [23]. The CAT activity was determined based on the rate of hydrogen peroxide disappearance, as indicated in the established methodologies with slight modifications [24,25] and adaptations for microplate analysis. An amount of 5 µL of the crude extract was placed with 295 µL of a H_2_O_2_ solution (11 mM, prepared in phosphate buffer) in a UV microplate (Corning 3635, Sigma Aldrich, St Louis, MO, USA). The rate of H_2_O_2_ disappearance was monitored in kinetic mode at an absorbance of 240 nm for 10 min with 45 s intervals at 37 °C (Biotek Epoch 2 microplate reader, Bio Tek Instruments Inc. Winooski, VT, USA). The blank (phosphate buffer) was subtracted from each absorbance, and the graphical method was used to determine the slope of each sample with these values. The molar extinction coefficient of H_2_O_2_ (39.5 L/mol/cm) was used to calculate the enzyme activity. This was expressed in U/mg, where each unit represents the amount of enzyme necessary to decompose 1 µmol of H_2_O_2_ per minute.

SOD activity was determined using a method that evaluates the autoxidation of pyrogallol in a base medium, with modifications to the original procedure [26,27]. A total of 7.5 µL of extract with 7.5 µL of 10 mM pyrogallol (prepared in ethanol) and 285 µL of tris–HCl buffer (50 mM) was placed in a UV microplate (Corning 3635). The absorbance was monitored in kinetic mode at 340 nm for 8 min at 37 °C (Biotek Epoch 2 microplate reader). Sodium phosphate buffer was used as a blank. The results were expressed in U/mg, where each unit represents the amount of enzyme required to inhibit pyrogallol autooxidation by 50%.

GPx activity was evaluated using a method that determines the NADPH oxidation rate, with tertbutyl hydroperoxide used as the substrate. The protocol was adapted for microplate analysis [21,28]. A total of 4 µL of each extract was placed in a microplate with 16 µL of sodium phosphate buffer (40 mM). Then, 100 µL of assay medium (100 mM potassium phosphate buffer (pH 7.0) in 1 mM EDTA), 20 µL of glutathione reductase (0.24 U/mL), and 20 µL of reduced glutathione (10 mM) were added, and this mixture was incubated for 10 min at 37 °C. Then, a 20 µL solution of NADPH (1.5 mM in 0.1% NaHCO_3_) was added and incubated at the same temperature for 3 min. Finally, 20 µL of tertbutyl hydroperoxide (12 mM) was added, and the absorbance was monitored in kinetic mode at 340 nm for 10 min (Biotek Epoch 2 microplate reader). Phosphate buffer was used as a blank. The molar extinction coefficient of NADPH (6.22 mM^−1^ cm^−1^) was used to calculate the results and expressed in U/mg. Each unit reflects the amount of enzyme required to oxidize 1 µmol NADPH/min.

### 2.5. Histomorphometric Analysis of Intestinal Epithelium

Immediately after evisceration, a longitudinal section of approximately 5 cm was taken from the duodenum and another from the jejunum of 5 animals per treatment. The duodenum and jejunum segments were taken 10 cm and 40 cm after the pylorus, respectively. Then, two cross-sections with different thicknesses (1 cm and 2 cm) were made on each segment. The 1 cm sections of the samples were washed and stored in 10% formalin, while the 2 cm thick samples were cut longitudinally, spread on a wooden base, and stored in 10% formalin for 3 days for proper tissue fixation. Subsequently, both sections were included in paraffin. A HM325 microtome was used to cut 4 µm-thick cross-sections (Thermo Fisher Scientific, Waltham, MA, USA), which were stained with hematoxylin and eosin (Sigma-Aldrich, St Louis, MO, USA) [29,30]. Ten measurements were taken for each variable for each segment of the intestine (duodenum and jejunum). These segments were visualized with a 10× objective and the Image View 2019 software.

The intestinal villus length (the distance from the apex to the lamina propria), width (the base, midpoint, and apex height), crypt depth (the entrance to the basal zone), and villus length–crypt depth ratio (V–C) were determined. The results are expressed in µm.

### 2.6. Statistical Analysis

In the analysis, each animal was considered as an experimental unit. The normality of all response variables was verified using the Kolmogorov test. A 2 × 2 factorial arrangement with a completely randomized design was used for the analysis of variance (ANOVA) of the antioxidant enzyme activity in the muscles and the histomorphometric analysis of the intestinal epithelium. The model considered the fixed effects of additives (FA or GP) and their interaction (FA × GP). Tukey’s test was used for comparison of means when significant differences between treatments were detected. The effect of the treatments was considered significant when *p* < 0.05, and trends were considered at *p* < 0.10.

The data were analyzed using the NCSS statistical software (version 2020, NCSS, Kaysville, UT, USA).

## 3. Results

### 3.1. Identification of Phenolic Compounds in Supplemented Grape Pomace

Supplementary Table S2 shows the retention times (Rt) and maximum absorption lengths for the phenolic acid and flavonoid standards used. Meanwhile, the spectral characteristics of the PCs identified in the grape pomace meal and fractions of phenolic acids and flavonoids (280 nm) are presented in Table 1 and Table 2.

The chromatograms of the PCs in Tempranillo GP obtained at 280 are shown in the Supplementary Materials (Figures S1–S5). Tempranillo GP contains approximately 22 phenolic compounds. It was estimated that this by-product would consist mainly of conjugated flavonoids (68%), 22% phenolic acids, and an approximate 9% proportion of stilbenes (monomers and dimers). Gallic (Tr 6.99 min) and protocatechuic acids (Tr 10.049) were identified as the primary hydroxybenzoic acids in this grape pomace (Figure S5). Caffeic acid and p-coumaric acid were also detected by spiking with the respective standards (Figure S3).

The spectral characteristics of the identified flavonoids reflect that most of these compounds were found in glycosylated form as derivatives of quercetin, myricetin, and rutin (Table 1 and Table 2). Similarly, the flavonols catechin (Tr 17.47 min) and epicatechin (Tr 17.66 min) were also identified as major compounds in this agro-industrial by-product. Meanwhile, when a mixture of the standards was added to the grape pomace sample, a slight response was observed in compounds of biological interest, such as rutin (22.36 min) and resveratrol (Tr 27.14 min), as shown in the Supplementary Materials (Figure S4). Table 3 shows the concentrations of phenolic compounds previously identified in the phenolic acids, flavonoids, and wash fractions, showing higher concentrations of epicatechin, catechin, and gallic acid.

### 3.2. Measurement of Antioxidant Enzyme Activity in Muscles

Table 4 shows the activity of the antioxidant enzymes CAT, SOD, and GPx, which were determined in the *Longissimus thoracis* muscle of pigs supplemented with FA and GP. The FA × GP interaction and FA did not affect antioxidant enzyme activity (*p* > 0.05). Only GP supplementation had an effect (*p* < 0.05). The results demonstrate that the inclusion of GP in finishing diets increased CAT activity by up to two times, while SOD and GPx activity increased by up to three times compared with the control and FA groups (*p* < 0.05).

### 3.3. Qualitative Visual Evaluation of Intestinal Morphology

**Figure 1:**
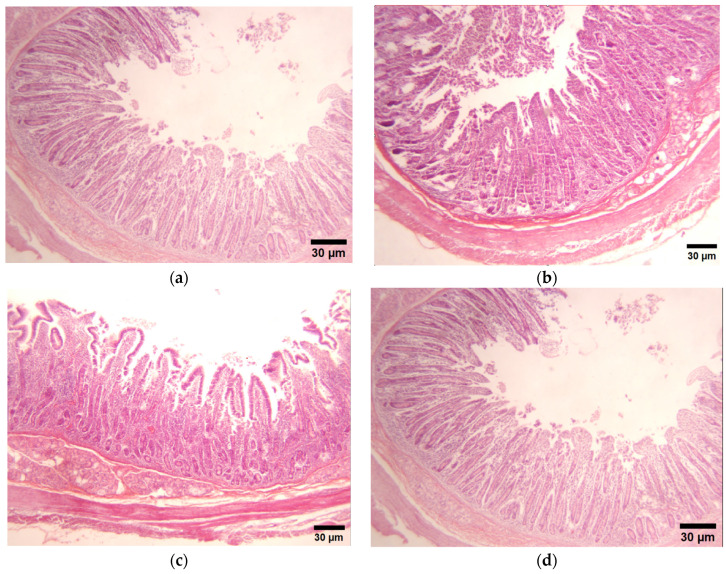
Histological section (duodenum) of pigs supplemented with grape pomace and ferulic acid. (**a**) Control (basal diet); (**b**) Ferulic acid (25 mg/kg); (**c**) Grape pomace (2.5%); (**d**) MIX: Ferulic acid (25 mg/kg) + GP (2.5%).

Figure 1 shows the morphological changes in the villi and intestinal lining of the duodenum and jejunum in pigs supplemented with both treatments. Compared with the control group, the intestinal epithelium in both segments exhibited highly heterogeneous villi in terms of height and width, with the duodenum displaying a greater villus length than the jejunum. Additionally, the control samples showed a lack of an epithelial lining and a clear delimitation of the intestinal lamina propria. In contrast, pigs supplemented with FA demonstrated more uniform villi, with a consistent length and width from the base to the midsection, tapering at the tip to form a diamond-like shape. These improvements were noted in both intestinal segments, though they were more pronounced in the jejunum.

Similarly, the GP treatment resulted in significant morphological changes, particularly in the width of the villi in both the duodenum and jejunum, distinguishing them from the other three treatments. The villi maintained a uniform width from base to tip, giving them a rectangular appearance. This group also exhibited reduced detachment of the intestinal epithelium and enhanced integrity of the intestinal lamina propria, with an increase in villus length compared with the control group.

### 3.4. Histomorphometric Parameters in Duodenum and Jejunum of Pigs Supplemented with FA and GP

Table 5 shows the effect of the supplementation of FA and GP on the histomorphometric parameters in the duodenum and jejunum of finishing pigs. In these intestinal segments, there was a significant interaction between both additives evaluated (*p* < 0.05) for the variables’ height, the base width of intestinal villi, and the V–C ratio. For the duodenum, with the individual inclusion of FA, increases of 35% and 32% in the height of intestinal villi were observed compared with the control and MIX groups, respectively. However, the individual addition of GP increased the base width of intestinal villi by 27% compared with the control (*p* < 0.05). Furthermore, the tip width of the intestinal villi increased by up to 12% with GP supplementation, and the villus–crypt ratio improved by 41% with the addition of FA compared with the control (*p* < 0.05).

For the jejunum, the FA × GP interaction was significant only for the height and base width of the intestinal villi. Pigs supplemented individually with FA or GP were 22% and 18% higher (*p* < 0.05) in villus height than the control, respectively (Table 5). Pigs supplemented individually with FA or GP had 22% and 18% greater villus height than the control group, respectively. Likewise, the tip width of the FA and GP groups was 27% and 17% greater than that observed in the control group, respectively (*p* < 0.05). Similarly, the GP increased (*p* < 0.05) the tip width in this intestinal segment (22%).

## 4. Discussion

### 4.1. Identification of Phenolic Compounds in Grape Pomace Tempranillo Variety

Tempranillo grape pomace exhibits a diverse profile of PCs, as indicated by the chromatograms and data provided in the Supplementary Materials. This diversity of PCs, both in spectral and structural characteristics, gives the by-product various bioactive properties (antioxidant, anti-inflammatory, antibacterial, antiviral, and anticancer properties, among others) that have been reported for these compounds present in grape pomace [12,31]. This study demonstrated that compounds such as gallic acid, epicatechin, catechin, quercetin, protocatechuic acid, and anthocyanins (not identified with a standard) predominate in this by-product. It has even been reported that protocatechuic acid represents one of the main active metabolites of polyphenols such as anthocyanins [31,32]. These PCs are of great interest—particularly for their antioxidant capacity—making them an attractive ingredient as a functional additive for monogastric diets.

Similarly, possible synergistic interactions between the PCs present enhance these effects at the physiological level. However, antagonistic interactions have also been reported between some PCs, such as gentisic acid with syringic acid and vanillic acid with gallic acid [33,34]. In general, most of their beneficial effects in monogastric diets are mainly attributed to proanthocyanidins, procyanidins, quercetin, catechin, resveratrol, gallic acid, protocatechuic acid, and chlorogenic acid [11,32,35]. In turn, in our study, we corroborated that most of these compounds are present in the GP Tempranillo variety and may be associated with the results of intestinal histomorphology and enzymatic activity, as well as in productive performance and carcass quality, as presented in our previous publication [16]. However, it is necessary to complement these results with the quantification of other major PCs (glycosylated derivatives and anthocyanins) such that the optimal doses of these metabolites in finishing pig diets can be established based on these concentrations. Likewise, it is pertinent to carry out in silico studies in the future to identify possible interactions between the main FCs and, thus, establish a possible mechanism of action with this type of complex matrix.

### 4.2. Antioxidant Enzyme Activity in Longissimus thoracis Muscle of Pigs Supplemented with FA and GP

The inclusion of GP in finishing diets improved the enzymatic activities of CAT, SOD, and GPx in the *Longissimus thoracis* muscle. Generally, these antioxidant enzymes prevent the formation of or neutralize reactive oxygen species (ROS) in cells by converting either superoxide radicals to H_2_O_2_ or H_2_O_2_ to O_2_ and H_2_O, and regulating processes involved in cell regeneration [36]. The results obtained in this study agree with previous research, showing that the inclusion of different PCs in monogastric diets activates the organism’s endogenous antioxidant defense [21]; therefore, cellular enzymatic activity is improved and helps to counteract oxidative stress caused by a specific stimulus [37,38]. Similarly, such a trend has been observed with matrices rich in these secondary metabolites, such as *Morus alba*, apple polyphenols, and grape extracts [39,40,41]. In this regard, it has been proposed that these changes exerted by PCs (quercetin, chlorogenic acid, resveratrol, ferulic acid, and proanthocyanidins) on enzymatic activity in pigs are related to a transition of muscle fibers toward a more oxidative phenotype [42,43], in which several signaling pathways that enhance antioxidant capacity (AMPK/SIRT1/PGC-1α, and NRF1/CaMKKKβ) are involved in pigs [44,45]. Generally, these types of oxidative fibers have a higher mitochondrial volume and SOD and CAT activity, unlike glycolytic phenotypes [36,42,46]. This information indicates that oxidative and oxidative–glycolytic (intermediate) muscles possess natural protection from the adverse effects of different free radicals. However, the onset of apoptosis or cell death and multiple factors (type of muscle, species, breed, feeding system, and time of exposure to stressors) will determine the initial and postmortem enzymatic activity [37,38,47]. This can be considered a fundamental limitation of this study; in particular, considering the availability of samples to analyze together with other response variables that could give greater robustness to the work (hydrogen peroxide content, lipid peroxidation, energy metabolites, and protein expression, among others).

### 4.3. Visual and Histomorphometric Evaluation of Intestinal Epithelium in Duodenum and Jejunum of Finishing Pigs Supplemented with FA and GP

Most digestion and absorption processes typically occur in the small intestine. The duodenum is the site of major chemical digestion via pancreatic secretions. Furthermore, the jejunum is considered the primary site of nutrient absorption [48]. However, the constant exposure of pigs to heat stress conditions (acute or chronic) has been associated with a loss of intestinal villi, which exposes the lamina propria and promotes intestinal permeability [6]. In this sense, based on a visual evaluation of the intestinal epithelium, relevant morphological changes in the villi were identified in both portions of the intestine, and characteristics of integrity, uniformity, and abnormalities were observed. This information is considered key and indispensable in complementing the results of the respective histological measures. With this visual evaluation, less detachment of intestinal villi was observed in the epithelium of pigs supplemented with FA and GP. Likewise, in both treatments, the lamina propria remained delimited, unlike the control group, which reflected an improvement in intestinal integrity [29]. Therefore, the observed changes in the epithelium suggest a protective effect of supplementing PC-rich sources on intestinal integrity.

Moreover, with the measurement of histomorphometric parameters, it was demonstrated that the individual inclusion of GP and FA significantly increased the width and length of the intestinal villi, respectively, in both the duodenum and the jejunum. In turn, the villus–crypt ratio increased in the duodenum of pigs supplemented with FA. In previous studies in rats and pigs supplemented with phenolic compounds, these favorable changes in villi have been associated with an improvement in the integrity of the intestinal epithelium through the modulation of tight junctions and increased functionality of intestinal barrier proteins [11,49] which, together with the villi’s increased surface area, can translate into increased nutrient absorption [50,51]. The beneficial results on intestinal villus characteristics in this study could be associated with the improved average daily gain (ADG) and feed efficiency of the animals. However, the results of our previous study [16] indicated that only a 10% (non-significant) increase in ADG was observed with GP supplementation. Previous studies have demonstrated that the inclusion of 6% GP in finisher pig diets also increases the height of intestinal villi in both the ileum and colon, along with the expression of tight-junction proteins (occludin-1, claudin-1, and zonula occludens-1), unlike the control group [52]. Similarly, the same trends have been observed in the duodenum and jejunum of weaned pigs supplemented with various PCs (vanillic acid, chlorogenic acid, protocatechuic acid, ferulic acid, and resveratrol, among others) under conditions of oxidative stress and various stressors [50].

The results of our research suggest that matrices rich in PCs, such as GP, and pure compounds, such as FA, could exert a protective function on the intestinal mucosa. In the case of GP, the profile and concentration of PCs will determine this beneficial effect. This protective function has been mainly attributed to the group of hydrolyzable tannins (HTs) and flavonoids (quercetin and catechins), as indicated in other studies [53,54]. These authors demonstrated that HT supplementation (1–3%) in finishing pigs increases the height, villus perimeter, villus–crypt ratio, and intestinal mucosal thickness in the duodenum segment, while a reduction in cell proliferation was observed along with the number of apoptotic cells in the cecum and colon [53]. Their findings are attributed to the formation of thin denatured protein films coating the mucous membrane of the intestinal walls and exhibiting selective antimicrobial properties [55].

Similarly, the improvements observed in histomorphometric variables could be explained by intestinal cell renewal processes, illustrated by the relationship between cell proliferation and apoptosis, which have been reported in other studies [48,53]. Even improvements in antioxidant activity (SOD, CAT, and GPx), together with the expression of tight-junction proteins [11], the modulation of the intestinal microbiota, and the anti-inflammatory properties exerted by PCs, have been proposed as the main mechanisms of action that explain the significant changes in intestinal barrier function. In turn, it has been proposed that the inclusion of secondary metabolites in monogastric diets as functional additives can activate type 1 and 2 receptors (T1R2/3) in the intestine, where glucagon peptide release takes place [6,56], and improve intestinal histomorphology variables under caloric stress conditions. Although this study showed remarkable changes in the duodenum and jejunum segments, in order to confirm its protective effect against temperature stress stimuli, it is advisable to evaluate additional variables in the intestine, such as transepithelial resistance, antioxidant enzyme activity, mucosal thickness, and tight-junction proteins, among others. Even in this study, it was expected to have more hostile conditions, typical of the region, which partially limited the experimental development. The experimental area of this study is subject to extreme humidity and temperature fluctuations that can promote severe damage to the intestinal epithelium of the finishing pigs. Nevertheless, this investigation suggests that using PC-rich sources as functional additives in monogastric diets could attenuate the impacts of heat stress on productive performance and intestinal health.

## 5. Conclusions

This research validated the potential of individual doses of ferulic acid (25 mg/kg) and grape pomace (2.5%) as rich sources of various phenolic compounds (gallic acid, protocatechuic acid, catechin, epicatechin, and glycosylated derivatives of myricetin, quercetin, and rutin) to improve some morphometric characteristics of intestinal villi. The synergistic effect of supplementation with both additives was observed to improve several characteristics of intestinal villi. Our research findings show that the inclusion of sources which are rich in proanthocyanidins and possess a diverse flavonoid profile, such as GP, improves the antioxidant capacity in postmortem muscle (SOD, CAT, GPx activity). Future research could continue this exploration through metabolomic and postmortem proteolysis studies, with which a detailed explanation could be provided for the information obtained in this study. The results support using these compounds as unconventional alternatives in animal feeding, highlighting their phytogenic and zootechnical benefits. This strategy, based on the bioactive compounds of plants, represents a promising approach to maintaining good productive performance and antioxidant status, especially in animals exposed to extreme environmental conditions.

## Figures and Tables

**Table 1:** Phenolic compounds identified in the methanolic extract of grape pomace.

Retention Time (min)	Area (%)	Absorbance Maximum (nm)	Identification
7.13	10.66	230, 270	Gallic acid
10.04	1.29	254, 298	Protocatechuic acid
12.50	1.96	282, 230, 326 s, 214 ps	NI
12.53	2.03	230, 286, 330	NI
13.97	5.01	230, 278	Catechin
15.05	3.80	230, 282	Catechin
15.80	1.23	234, 254	NI
16.8	1.16	234, 282, 310 s	NI
17.52	5.31	234, 282, 318	NI
18.44	6.32	234, 274, 354 s	Glycosidic flavonoid
18.86	3.23	234, 278	Epicatechin
19.17	1.64	234, 278, 370	Glycosidic flavonoid
20.37	1.06	234, 278, 362	Rutin derivative
20.92	1.51	238, 274, 358	Epicatechin
21.05	1.02	238, 274, 358	Rutin glycosidic derivative
21.19	-	238, 278, 518	Anthocyanin
21.38	1.33	238, 278	Epicatechin
21.86	0.63	238, 278	Epicatechin
22.11	0.74	238, 278, 518	Anthocyanin
22.83	2.491	254, 302 s, 358	Rutin
23.03	4.311	234 s, 262, 390 s, 358	Quercetin derivative
23.61	0.09	220, 254, 298, 334 s, 414 s, 530	Anthocyanin
24.11	5.55	222, 214 h, 258, 530	Anthocyanin
25.34	0.76	222, 250, 322, 370 h, 574	Anthocyanin
34.47	---	226, 258, 278	NI

NI: Not identified.

**Table 2:** Phenolic compounds identified in phenolic acid and flavonoid fractions.

Retention Time (min)	Absorbance Maximum (nm)	Identification
	Phenolic Acid Fraction	
7.41	230, 270	Gallic acid
10.21	228, 258, 294	Protocatechuic acid
12.38	270, 338 s	NI
13.98	238, 278	NI
14.27	266	NI
15.97	262, 290, 358, 370	NI
16.99	242, 338	NI
17.64	238, 274	Catechin
19.57	234, 242, 254, 298 s, 366	NI
21.11	238, 278	Epicatechin
22.02	222, 266, 350, 538	Anthocyanin
22.39	222 s, 254,298 s, 366	NI
28.34	242, 248	NI
	Flavonoid Fraction	
23.8	238, 254, 266 s, 298 s, 358, 530	Anthocyanin
24.79	234, 242, 258, 278, 298 s, 350, 430 s, 530	Anthocyanin
24.91	222, 266, 298 s, 346, 530	Anthocyanin
25.19	238, 258, 310 s, 358	
26.53	246, 278	Catechin
26.60	250, 278	Epicatechin
26.96	242, 262, 370	Quercetin derivative
27.72	242, 278, 370	Quercetin derivative
30.21	298, 340 s, 534	Anthocyanin

NI: Not identified.

**Table 3:** Quantification of phenolic compounds in Tempranillo grape pomace at 280 nm.

	Standard	Concentration (µg/g)
1	Gallic acid	87.68
2	Protocatechuic acid	0.36
3	Catechin	79.58
4	Epicatechin	103.25
5	Quercetin	1.76

**Table 4:** Enzymatic activity in *Longissimus thoracis* muscle of pigs supplemented with FA and GP.

	Treatment						*p*-Value		
	FA, mg	0		25		SEM	FA	GP	FA × GP
	GP, %	0	2.5	0	2.5				
CAT, U/mg		0.043	0.088	0.037	0.077	0.012	0.506	0.009	0.860
SOD, U/mg		0.065	0.220	0.064	0.231	0.049	0.925	0.011	0.896
GPx, U/mg		0.006	0.023	0.008	0.023	0.005	0.800	0.010	0.830

FA: ferulic acid; GP: grape pomace; CAT: catalase; SOD: superoxide dismutase; GPx: glutathione peroxidase. SEM: standard error of the mean.

**Table 5:** Histomorphometric parameters of duodenum and jejunum of pigs supplemented with FA and GP.

Variable	Treatment						*p*-Value		
	FA, mg	0		25		SEM	FA	GP	FA × GP
	GP, %	0	2.5	0	2.5				
Duodenum									
Villus height (µm)		1705.8 a	1988 ab	2302.8 b	1740.2 a	68.46	0.090	0.160	0.001
Base width (µm)		386.4 a	491.5 b	451.1 ab	410.5 a	23.47	0.730	0.190	0.001
Medium width (µm)		448.04	551.7	479.1	494.9	23.65	0.767	0.112	0.326
Tip width (µm)		392.6	459.2	382.4	408.9	19.55	0.140	0.030	0.320
Crypt depth (µm)		1379.0	1284.6	1303.6	1366.9	0.05	0.940	0.739	0.110
V–C ratio		1.25 a	1.54 ab	1.77 b	1.27 a	0.00	0.130	0.229	0.001
Jejunum									
Villus height (µm)		1534.7 a	1818.7 b	1878.4 b	1528.9 a	134	0.840	0.810	0.030
Base width (µm)		359.4 a	483.2 b	336.4 a	375.0 a	19.6	0.001	0.001	0.046
Medium width (µm)		439.7	457.25	384.9	496.7	37.2	0.830	0.101	0.223
Tip width (µm)		334.1	398.9	282.4	358.0	24.2	0.069	0.010	0.825
Crypt depth (µm)		1133.5	1361.6	1322.7	1250.3	76.4	0.610	0.323	0.069
V–C ratio		1.39	1.37	1.438	1.23	0.12	0.836	0.316	0.547

FA: ferulic acid; GP: grape pomace. Means within a row with different letters indicate significant differences (*p* < 0.05). Trends (*p* < 0.1). SEM: standard error of the mean.

## Data Availability

The data presented in this study are available upon request from the corresponding author.

## Supplementary Materials

The following supporting information can be downloaded at https://www.mdpi.com/article/10.3390/ani15162382/s1, Table S1. Ingredients and chemical composition of experimental diets. Table S2. Retention times and absorption maximum of phenolic acid and flavonoid standards. Figure S1. HPLC chromatogram of methanolic extract of grape pomace and phenolic acids standards at 280 nm. Figure S2. HPLC chromatogram of methanolic extract of grape pomace, flavonoid, and resveratrol standards at 280 nm. Figure S3. HPLC chromatogram of grape pomace extract, flavonoid standards at 280 nm. Figure S4. HPLC chromatogram of grape pomace extract, flavonoid standards at 280 nm. Figure S5. HPLC chromatogram of phenolic acid and flavonoids fraction of grape pomace at 280 nm.
