# Supplementary material for: Grape Pomace and Ferulic Acid Improve Antioxidant Enzyme Activity and Gut Histomorphometry in Heat-Stressed Finishing Pigs
Source: Animals (Basel). 2025 Aug 13;15(16):2382. doi: 10.3390/ani15162382 (PMC12382960; doi:10.3390/ani15162382)
Supplement: Supplementary file 1 [file animals-15-02382-s001.zip › animals-3746115-supplementary.pdf]

animals

Supplementary Materials

Grape Pomace and Ferulic Acid Improve Antioxidant Enzyme Activity and Gut Histomorphometry in Heat-Stressed Finishing Pigs

María A. Ospina-Romero [1], Leslie S. Medrano-Vázquez [1,2], Araceli Pinelli-Saavedra [1], Miguel Ángel Barrera-Silva [2], Martín Valenzuela-Melendres [1], Miguel Ángel Martínez-Téllez [1], Reyna Fabiola Osuna-Chávez [2], María del Refugio Robles-Burgueño [1] and Humberto González-Rios [1,*]

Table S1. Ingredients and chemical composition of experimental diets.

Ingredients	Treatments [1]			
	Control	FA	GP	MIX
Wheat grain, %	76.2	76.2	73.7	73.7
Soybean meal, %	17.0	17.0	17.0	17.0
Vegetable oil, %	4.4	4.4	4.4	4.4
Premix [2], %	2.4	2.4	2.4	2.4
GPM, %	0.0	0.0	2.5	2.5
FA, mg/kg	--	25	--	25
Proximate Composition				
Crude protein, %	14.0	14.0	13.9	13.9
Moisture, %	11.9	11.9	11.9	11.9
Fat, %	7.0	7.0	7.0	7.0
Crude fiber, %	2.0	2.0	2.1	2.1
Ash, %	7.0	7.0	7.0	7.0
NFE [3], %	58.1	58.1	58.1	58.1
ME [4], Mcal/kg	3.35	3.35	3.34	3.34

[1] Control: animals receiving basal diet, DB without additives; FA: DB + 25 mg FA/kg; GP: BD + 2.5% GP/kg; and MIX: BD + 25 mg FA + 2.5% GP/kg). [2] Premix: Premix of amino acids, vitamins, and minerals. Each kilogram of feed provided 9.5 g dicalcium phosphate, 8.3 g limestone, 3.55 g sodium chloride, 2.3 g L-lysine, 0.5 g DL-methionine, 0.35 g L-threonine, 0.15 g L-tryptophan, 80 mg DL-tocopherol acetate, 2.2 g retinol acetate, 16.5 mg cholecalciferol, 4.4 mg sodium bisulfite, 242 mg choline, 33 mg niacin, 8.8 mg riboflavin, 24.2 mg D-pantothenic acid, and 0.04 mg vitamin B12. [3] NFE: Nitrogen-free extract. [4] ME: Metabolizable energy, mega-calories per kg. Data reported by Ospina-Romero et al. [11].

 https://doi.org/10.3390/ani15162382

Table S2. Retention times and absorption maxima of phenolic acid and flavonoid standards.

	Standard	Retention Time (Rt, min)	Absorbance maximum wavelength (nm)
1	Gallic acid	6.99	(230, 270)
2	Protocatechuic acid	10.14	(254,298)
3	Gentisic acid	13.51	230, 298s, 330
4	Catechin	14.746	234, 280
5	Chlorogenic acid	16.15	226, ps290,326
6	Cafeic acid	16.63	234, ps298,322
7	Siringic acid	17.25	270
8	Epicatechin	17.66	234, 280
9	Vainillin	18.30	234,282,310
10	P-Coumaric acid	20.07	238,294s, 310
11	Ferulic acid	21.55	234, 298s, 322
12	Sinapic acid	21.83	234,326
13	Rutin	22.36	254,298h,354
14	Isoferulic acid	22.44	234,290s,294,322
15	O-Coumaric acid	23.487	234, 278, 326
16	Myricetin	25.94	234,254,302h,374
17	Resveratrol	27.14	234, 306, 318h
18	Trans-cinamic acid	29.463	278,234
19	Quercetin	30.87	238, 254,302h, 370

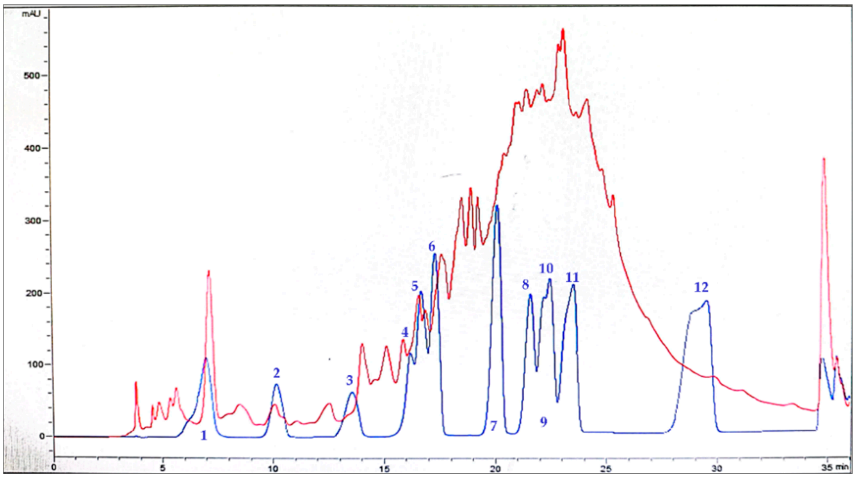

Figure S1. HPLC chromatogram of methanolic extract of grape pomace and phenolic acids standards at 280 nm. In blue: Standards of: 1. Gallic acid (Rt 6.99min); 2. Protocatechuic acid (Rt 10.14 min);3. Gentisic acid (Rt 13.51 min); 4. Chlorogenic acid (Rt 16.15 min); 5. Cafeic acid (Rt 16.63min); 6. Siringic acid (Rt 17.25 min); 7. P-coumaric acid (Rt 20.07 min); 8. Ferulic acid (Rt 21.55); 9. Sinapic acid (Rt 21.83); 10. Isoferulic acid (Rt 22.44); 11. O-Coumaric acid (Rt 23.48 min); 12. Transcinamic acid (29.46 min). In red: Methanolic extracts of grape pomace.

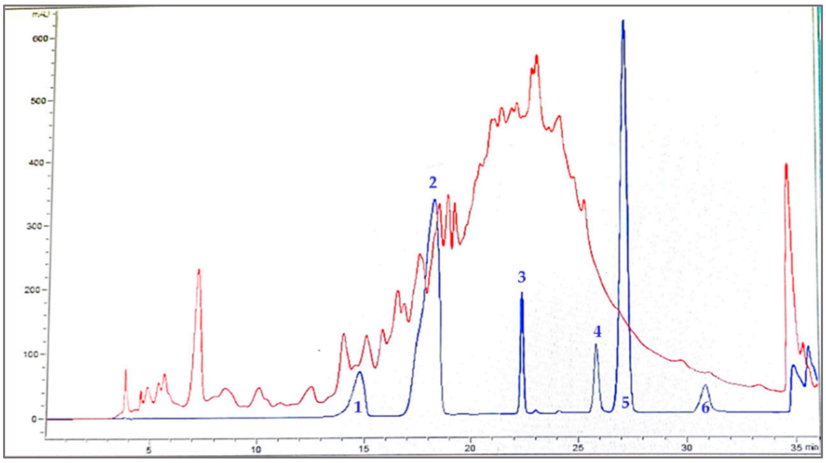

Figure S2. HPLC chromatogram of methanolic extract of grape pomace, flavonoid, and resveratrol standards at 280 nm. In blue: Standards of: 1. Catechin (Rt14.74 min); 2. Epicatechin (Rt 17.66 min);3. Rutin (Rt 22.36 min); 4. Myricetin (Rt 25.83 min); 5. Resveratrol (Rt 27.14min); 6. Quercetin (Rt 30.87 min). In red: Methanolic extracts of grape pomace.

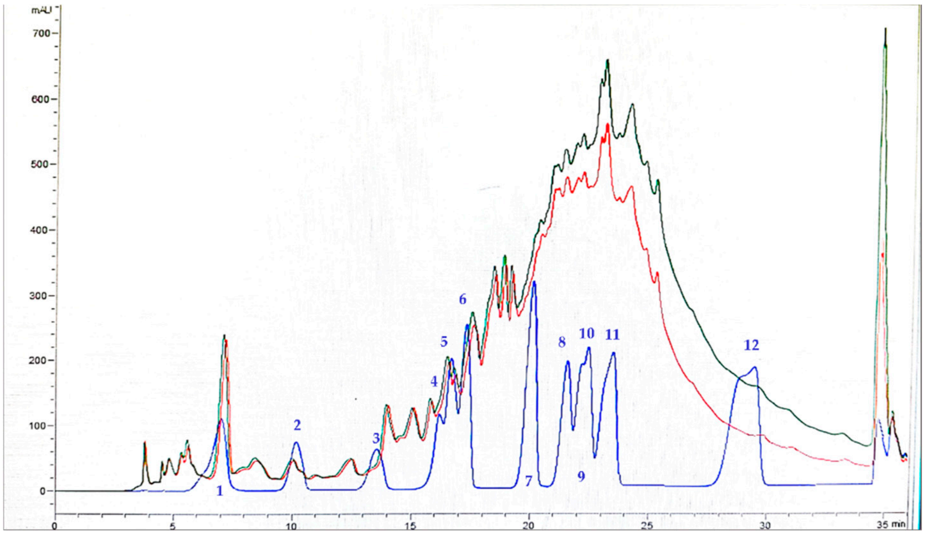

Figure S3. HPLC chromatogram of grape pomace extract, flavonoid, and resveratrol standards at 280 nm. In blue: Standards of: 1. Gallic acid (Rt 6.99min); 2. Protocatechuic acid (Rt 10.14 min);3. Gentisic acid (Rt 13.51 min); 4. Chlorogenic acid (Rt 16.15 min); 5. Cafeic acid (Rt 16.63min); 6. Siringic acid (Rt 17.25 min); 7. P-coumaric acid (Rt 20.07 min); 8. Ferulic acid (Rt 21.55); 9. Sinapic acid (Rt 21.83); 10. Isoferulic acid (Rt 22.44); 11. O-Coumaric acid (Rt 23.48 min)**;** 12. Transcinamic acid (29.46 min). In red: Methanolic extract of grape pomace. In green: Methanolic extract of grape pomace spiked with phenolic acid standards.

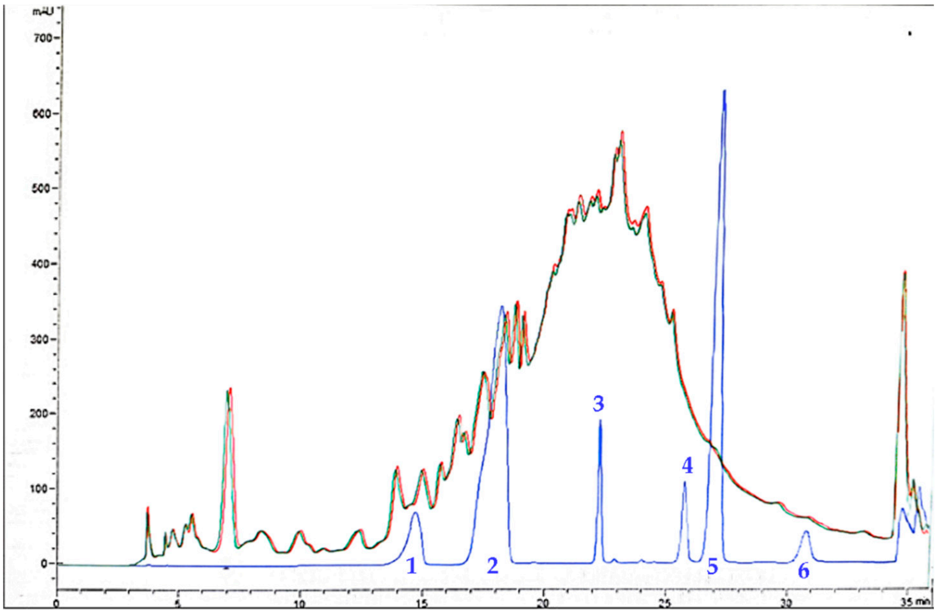

Figure S4. HPLC chromatogram of grape pomace extract, flavonoid, and resveratrol standards at 280 nm. In blue: Standards of: 1. Catechin (Rt14.74 min); 2. Epicatechin (Rt 17.66 min); 3. Rutin (Rt 22.36 min); 4. Myricetin (Rt 25.83 min); 5. Resveratrol (Rt 27.14min); 6. Quercetin (Rt 30.87 min). In red: Methanolic extract of grape pomace. In green: Methanolic grape pomace extract spiked with flavonoids and resveratrol standards.

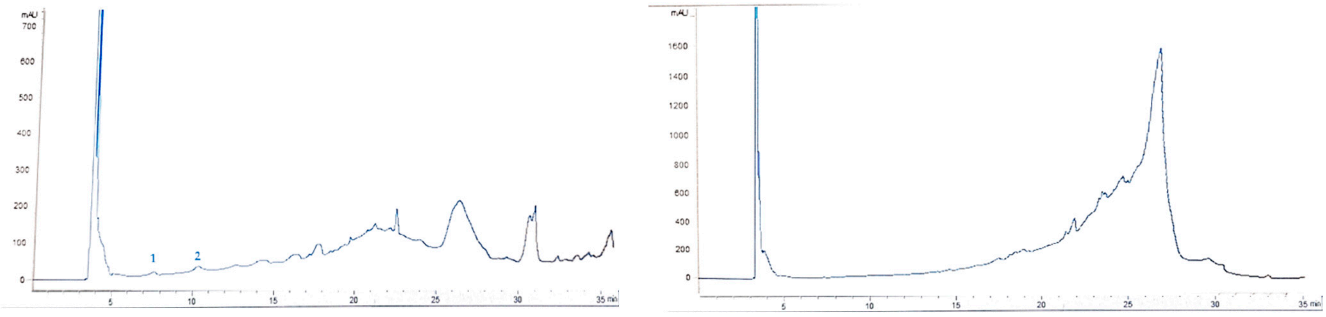

(**a**) (**b**)

Figure S5. HPLC chromatogram of phenolic acid and flavonoids fraction of grape pomace at 280 nm. (**a**) Phenolic acid fraction: PCs identified 1. Gallic acid; 2. Protocatechuic acid. (**b**) Flavonoids Fraction
